# Sexual complications of penile frature in men who have sex with men

**DOI:** 10.1590/S1677-5538.IBJU.2017.0520

**Published:** 2018

**Authors:** Rodrigo Barros, Gabriel Lacerda, Alex Schul, Paulo Ornellas, Leandro Koifman, Luciano A. Favorito

**Affiliations:** 1 Hospital Municipal Souza Aguiar, Rio de Janeiro, RJ, Brasil; 2 Universidade Estadual do Rio de Janeiro (UERJ), RJ, Brasil; 3 Hospital Federal da Lagoa, Rio de Janeiro, RJ, Brasil

**Keywords:** Penis, Homosexuality, Coitus

## Abstract

**Objectives:**

Evaluate the demographic data, etiology, operative findings and results of surgical treatment of penile fracture (PF) in men who have sex with men(MSM) with emphasis on sexual complications.

**Materials and Methods:**

We studied 216 patients underwent surgical correction of PF at our hospital. Patients self-identified as MSM were followed for at least 6 months. Demographic data, presentation, operative findings, International Index of Erection Function - 5 (IIEF-5) and the Premature Ejaculation Diagnostic Tool.

**Results:**

Of 216 PF cases, 4 (1.8%) were MSM. All cases resulted from sexual activity and all patients reported using the “doggy style” position during anal intercourse. Unilateral or bilateral injury of corpus cavernosum was found in 2 patients each. One (25%) patient had complete urethral injury associated with bilateral corpus cavernosum lesion. During the follow-up period, all patients developed some type of sexual complication. One patient reported penile pain during intercourse. Another patient experienced low sexual desire and premature ejaculation. This patient was also dissatisfied with the aesthetic result of the surgical scar and complained about decreased penis size after surgery. The third case developed delayed ejaculation. The fourth patient experienced mild to moderate erectile dysfunction. This same patient presented with penile curvature. Finally, palpable fibrotic nodules in the operative area were observed in all cases.

**Conclusions:**

Sexual activity in the “doggy style” position was the commonest cause of PF in MSM. Sexual dysfunction is always present in gay man after surgery for PF. However, additional studies with larger samples should be coinducted.

## INTRODUCTION

Penile fracture (pf) is an uncommon form of urologic trauma. Sexual intercourse is the com-monest cause of fracture of the penis (1). Immediate surgical exploration is the current standard treatment and aims at restoring the anatomical and functional integrity of the penis. However, this approach is not free from complications and sexual dysfunctions can occur (2).

Determining the number of men who have ever had sex with another man (msm) is difficult. In a study conducted in the united states, at least 5% of men, reported having ever had sex with men (3). This population is also at a risk of pf. Nevertheless, most studies on pf have only included heterosexual patients. Moreover, msm have received limited attention in sexual medicine li-terature and non-heterosexual orientation is an exclusion criterion in many large scale studies in sexual medicine (4, 5). Moreover, the majority of instruments for the assessment of sexual problems have not been validated in homosexual patients (6). An internet-based survey with men who have sex with men (msm) found that 79% of men reported at least one sexual dysfunction symptom such as low sexual desire, erection problems, and performance anxiety (7). Accordingly, we believe that sexual dysfunction may be present in msm operated on for pf. The aim of this study is to evaluate the demographic data, etiology, operative findings and results of surgical treatment of pf in msm, with emphasis on sexual complications.

## MATERIALS AND METHODS

Between january 1997 and december 2016, 216 patients underwent surgical correction of pf at our hospital. Patients self-identified as msm were followed at the andrology outpatient clinic of this institution for at least 6 months.

Epidemiological and clinical presentation data and operative findings were reviewed retros-pectively using the medical records. All patients were submitted to the surgical technique utilized in our department, which consists of making a circular sub-coronal incision followed by further penile degloving and reconstruction of the corpus cavernosum and urethra, as necessary (8).

After the sixth postoperative month, patients were interviewed and questioned about any sexual dysfunction. The evaluation of postoperative erectile function was carried out by filling out the international index of erection function - 5 (iief-5) and the premature ejaculation diagnostic tool was used for the screening survey to assess risk of premature ejaculation. Minor modifications to syntax were made and specific terms for the subject's partner were replaced with gender neutral pronouns to adapt it for msm.

The study design was approved by the ethics and human research committee of the institution.

## RESULTS

Of 216 pf cases treated in our emergency room between january 1997 and december 2016, 4 (1.8%) were msm. Their age varied from 36 to 46 years old (mean, 41 years) and all patients were single. Time between trauma and surgery varied from 4 to 18 hours (mean, 12.7 hours). All cases resulted from sexual activity and all patients reported using the “doggy style” position during anal intercourse.

**Figure 1:**
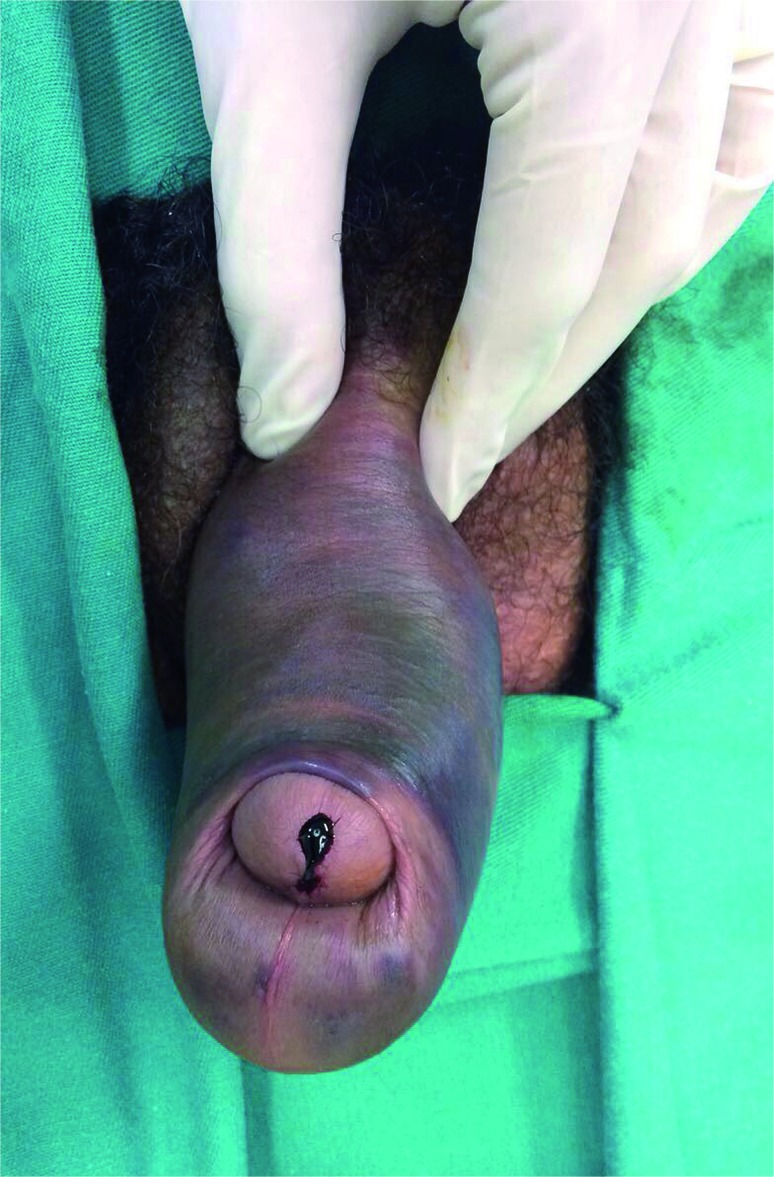
After a “doggy style” position the patient present penile fracture, we can observe a great hematoma and the typical aspect of eggplant deformity and meatal bleeding. During the surgery we identified a bilateral rupture of the CC and transection of the penile urethra.

With respect to the clinical presentation, all patients experienced immediate penile detumescence. Penile pain and a cracking sound during trauma were noticed by 3 (75%) patients. Extensive penile edema was observed in all cases. Only one (25%) patient experienced urethral bleeding and surgical exploration revealed urethral injury in this patient (Figure-1).

All patients were submitted to surgery after clinical diagnosis, without the need for complementary tests. With regard to operative findings, unilateral or bilateral injury of corpus cavernosum was found in 2 patients each. One (25%) patient had complete urethral injury asso-ciated with bilateral corpus cavernosum lesion.

Follow-up time varied from 6 to 16 months (mean, 10 months). During the follow-up period, all patients developed some type of sexual complication. It is worth noting that no patient had sexual problems before the trauma. One patient reported penile pain during intercourse. Another patient experienced low sexual desire and premature ejaculation. This patient was also dissatisfied with the aesthetic result of the surgical scar and complained about decreased penis size after surgery. The third case developed delayed ejaculation. The fourth patient experienced mild to mo derate erectile dysfunction (iief-5 score = 14) and need oral treatment with phosphodiesterase type 5 inhibitor (pde5) with a satisfactory response. This same patient presented with penile curvature. Intracavernous prostaglandin injection showed a curvature <30°. Penetration was not impaired and the treatment was conservative. Finally, palpable fibrotic nodules in the operative area were observed in all cases (Table-1).

**Table 1:** Demographic data, presentation, operative findings and sexual complications.

Patient	1	2	3	4
Age	36	45	46	37
Etiology	Anal intercourse/	Anal intercourse/	Anal intercourse/	Anal intercourse/
	“doggy style” position	“doggy style” position	“doggy style” position	“doggy style” position
Time between trauma and surgery (hours)	18	04	15	14
Presentation	Pain, cracking sound, detumescence and haematoma	Cracking sound detumescence, haematoma and urethral bleeding	Pain, cracking sound, detumescence and haematoma	Pain, detumescence and haematoma
Type of lesion	Unilateral corpus cavernosum	Bilateral corpus cavernosum and urethra	Bilateral corpus cavernosum	Unilateral corpus cavernosum
Sexual complications	Penile pain and fibrotic nodule	Premature ejaculation, low sexual desire, aesthetic dissatisfaction and fibrotic nodule	Delayed ejaculation and fibrotic nodule	Erectile dysfunction, penile curvature and fibrotic nodule

## DISCUSSION

There is evidence about the importance of genetic, autoimmune, and neurohormonal factors in the development of sexual orientation. Although homosexuality is widely established as a sexual orientation, the majority of religious authorities, as well as some political institutions, consider sex with people of the same gender unnatural (9). For this reason, many people are still unwilling to report their sexual orientation. Moreover, patients with pf may be too embarrassed to seek medical attention in the emergency room (10). This may explain why we found only 4 cases of pf in msm. In addition, despite our experience with 216 cases since 1997, all patients self-reported as msm only in the last year of this research and, one of these patients reported his sexual orientation at the third follow-up visit when his comfort level with the physicians increased.

Sexual intercourse is often associated with injury, especially if the sex act is more vigorous. Some studies reported that the “woman-on-top” position represented a major risk for pf, as the female partner usually controls the movements, and may inadvertently land the entire weight on the erect penis if it slips out of the vagina (11, 12). The same may occur in msm anal sex. However, it is unknown whether anal intercourse may be associated with an increased risk of pf. Reis et al. Found 4 homosexual patients in his sample of 42 cases of pf. Half of the patients reported being on top and the other half had intercourse in the “doggy style” position (11). In our recent study, we found that pf is most often caused by the “man-on-top” and “doggy style” positions. Moreover, these positions showed more associations with bilateral fractures of the corpus cavernosum and urethral lesions (1). In this study, all cases occurred during sexual activity when anal intercourse was being performed in the “doggy- style” position.

There has been limited investigation of sexuality and sexual dysfunction in non-heterosexual population and much of the medical literature on these sexual minority groups is centered on high-risk sexual behaviors and sexual dysfunction in hiv positive men (13). Despite this, there is evidence in the literature that psychological morbidity tends to be commoner in gays. Moreover, this can be due to secondary societal stigma against homosexuals (14, 15).

Research conducted by Bancroft et al. Evaluated a large sample of homosexual men and ma-tched heterosexual men for erectile dysfunction and ejaculatory problems. Erectile dysfunction is commoner in homosexual men with statistical significance and plays a more critical role in the sexual lives of homosexual men (16). According to Lau, et al., msm who experienced discrimination because of their sexual orientation were more predisposed to erectile dysfunction and premature ejaculation. Those msm who experienced anxiety because of their orientation were more likely to develop performance anxiety and sexual dysfunctions (15). In this study, we observe ejaculatory disturbances, low sexual desire secondary to pf in addition to aesthetic dissatisfaction and we believe they are linked to psychological aspects.

Hirshfield et al. Studied sexual dysfunctions in non-heterosexual men and found lower sexual drive (57%), erectile dysfunction (45%), performance anxiety (44%), lack of pleasure during sex (37%), inability to achieve orgasm (36%), premature ejaculation (34%), and pain during sex (14%). In addition, they noted that sexual problems were reported more frequently by men who were young, not in a relationship, and hiv-positive (7). A belgian study evaluating msm found an incidence of erectile dysfunction of 45% and the most common form of treatment was oral medi-cation (pde5). Most of the msm using such medication (83%) were satisfied with the result of the treatment (17). In our sample, one patient (25%) experienced erectile dysfunction and was treated with pde5 with a satisfactory response.

Reis et al. (11) did not correlate the complications found with sexual orientation, despite mentioning 4 cases of homosexuals operated on for pf in his sample. Thus, we were unable to find studies evaluating the development of sexual dysfunction especially in msm after the treatment of penile fracture. In this study, all patients self-identified as msm had some type of sexual complaint after surgery. These findings are different from those found in other heterosexual patients in our sample. Of 212 pf cases with different etiologies, 53 patients were properly followed up and 24 (45.2%) have developed sexual dysfunctions (ejaculatory dysfunction in 9.4%, low desire in 9.4%, penile curvature in 11.3%, erectile dysfunction in 16% and penile pain in 24.5%).

To our knowledge, the present study was the first to consider outcomes of pf in msm. Ne-vertheless, this study has limitations owing to the small number of cases and methodological weaknesses. The instruments that were modified have not been validated for the assessment of sexuality in this population and may introduce measurement bias. Moreover, we were unable to match heterosexual men with pf during anal intercourse for comparing the complications found in our sample.

## CONCLUSIONS

Fracture of the penis is a rare urological emergency and there is a lack of studies with msm. Sexual activity in the “doggy style” position was the commonest cause in this population. Sexual dysfunction is always present in non-heterosexual men after surgery for pf. However, additional studies with larger samples should be conducted.
